# Renoprotective effects of SGLT2 inhibitors in patients with Fabry disease

**DOI:** 10.1016/j.ymgmr.2025.101271

**Published:** 2025-10-17

**Authors:** Hayaki Okamoto, Shunsuke Goto, Mika Fujita, Hideki Fujii

**Affiliations:** Division of Nephrology, Kobe University Graduate School of Medicine, Kobe, Japan

**Keywords:** Fabry disease, Sodium–glucose co-transporter 2 inhibitors, Chronic kidney disease, eGFR slope

## Abstract

**Background:**

Fabry disease (FD) is a rare X-linked lysosomal storage disorder characterized by globotriaosylceramide (Gb3) accumulation, resulting in kidney and cardiac dysfunction. Although enzyme replacement therapy (ERT) and chaperone therapy are the standard therapies, progression of renal decline persists. Sodium–glucose co-transporter 2 (SGLT2) inhibitors exert renoprotective effects in chronic kidney disease (CKD), but their efficacy in FD remains unknown.

**Methods:**

We retrospectively analyzed data of 10 patients with FD treated with SGLT2 inhibitors and compared their renal outcomes to 18 patients with CKD without FD. The estimated glomerular filtration rate (eGFR) slope, urinary albumin-to-creatinine ratio (UACR), and plasma brain natriuretic peptide (BNP) levels were assessed 1 year before and after initiating SGLT2 inhibitor therapy. Linear mixed-effects models were employed for statistical analysis.

**Results:**

In patients with FD, the annual eGFR decline significantly improved from −4.38 mL/min/1.73 m^2^/year (IQR: −10.57 to 0.59) before treatment to 1.25 (IQR: −4.16 to 9.74) after treatment (*p* < 0.05). This improvement remained significant after adjusting for confounding factors. In contrast, the annual eGFR decline in patients with CKD without FD also tended to improve, albeit without significance. Notably, the initial eGFR decline usually seen with SGLT2 inhibitors in CKD was not observed in the FD cohort. UACR and plasma BNP levels remained unchanged after SGLT2 inhibitor therapy.

**Conclusions:**

SGLT2 inhibitors substantially attenuated the decline in eGFR in patients with FD. These findings support their potential as a renoprotective adjunct in the management of FD.

## 1 Introduction

Fabry disease (FD) is a rare X-linked congenital metabolic disorder caused by mutations in the alpha-galactosidase A gene, which encodes the lysosomal enzyme α-galactosidase A. These mutations result in deficient enzymatic activity, resulting in the systemic accumulation of globotriaosylceramide (Gb3), which is a substrate of α-galactosidase A, in various tissues. Kidney, heart, and cerebrovascular dysfunction are the primary clinical manifestations in FD and are major contributors to morbidity and mortality in affected individuals [1,2]. Enzyme replacement therapy (ERT) and pharmacological chaperone therapy, which are the standard treatments for FD, slow the progression of kidney and cardiac involvement, thereby improving life expectancy [1,2]. However, end-stage kidney disease or advanced cardiac failure still occur in some cases despite these interventions [3,4]. Although adjunctive therapies, such as angiotensin-converting enzyme inhibitors (ACE-Is) or angiotensin II receptor blockers (ARBs), are commonly used, their therapeutic efficacy in FD remains limited [1]. Furthermore, while paricalcitol, an active vitamin D receptor activator, reportedly reduces urinary protein in patients with FD [5], its clinical efficacy remains unclear.

Sodium–glucose co-transporter 2 (SGLT2) inhibitors, which were developed as therapeutic agents for diabetes mellitus, have been used in the management of chronic kidney disease (CKD) and heart failure even in patients without diabetes [6]. The renoprotective and cardioprotective effects of SGLT2 inhibitors may involve various nonglycemic mechanisms such as reducing inflammation and oxidative stress, exerting antifibrotic properties, modulating energy metabolism, and reducing kidney interstitial volume [7,8]. Thus, SGLT2 inhibitors may also provide similar therapeutic benefits for kidney and heart dysfunction in patients with FD. However, clinical evidence supporting their efficacy in FD remains scarce, and their therapeutic utility in this population remains unknown.

This retrospective case-control study aimed to explore the effects of SGLT2 inhibitors on estimated glomerular filtration rate (eGFR) slopes, the urinary albumin-to-creatinine ratio (UACR), and plasma B-type natriuretic peptide (BNP) levels in patients with FD using data derived from our institutional database.

## 2 Materials and methods

### 2.1 Study design and population

**Fig. 1:**
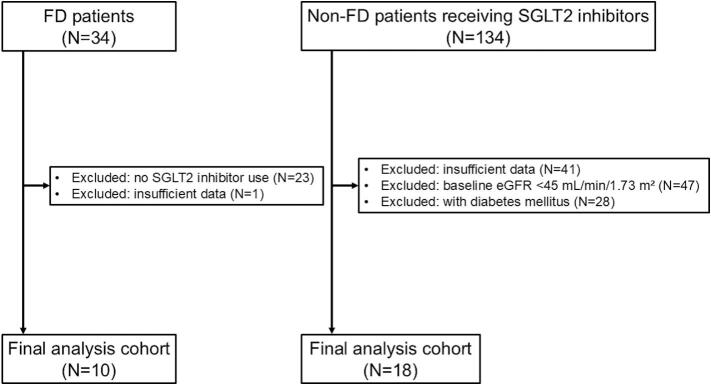
Flowcharts of study selection. FD, Fabry disease; eGFR, estimated glomerular filtration rate; SGLT2, sodium–glucose co-transporter 2.

This retrospective study utilized data from our institutional database. The cohort consisted of 34 consecutive patients with FD (14 men and 20 women) who were followed at our division between January 2008 and December 2024. Of these, 23 patients who did not receive SGLT2 inhibitors were excluded, and one patient was excluded due to insufficient eGFR data, i.e., lacking eGFR measurements spanning at least 3 months before and after initiation of SGLT2 inhibitor therapy. Finally, 10 patients with FD were included in our analysis (Fig. 1).

To compare the changes in eGFR following SGLT2 inhibitor therapy between patients with and without FD, we included a cohort of patients with CKD without FD who received SGLT2 inhibitors. For the cohort, we selected 134 consecutive patients who were followed at our division in December 2024. Of these, patients were excluded if they lacked eGFR data spanning at least 3 months before and after initiating SGLT2 inhibitor therapy (*N* = 41), had an eGFR of <45 mL/min/1.73 m^2^ (*N* = 47), or had diabetes mellitus (*N* = 28). Finally, a total of 18 patients were analyzed in this study (Fig. 1).

Clinical information, including age, sex, hypertension, diabetes mellitus, hyperlipidemia, FD genotype, FD-specific medications, renin-angiotensin system (RAS) inhibitors, eGFR, UACR, plasma BNP, and alfa-galactosidase A activity, was obtained from patient medical records. The eGFR was calculated using the Japanese-specific equation for GFR estimation [9].

To assess the effects of SGLT2 inhibitors in patients with FD, we evaluated alterations in kidney and cardiac parameters, including eGFR, UACR, and plasma BNP, during the 1-year period before and after treatment initiation. Kidney involvement was assessed using the eGFR and the UACR, while plasma BNP levels were employed as a marker of cardiac involvement. Data obtained during hospitalization were excluded because various factors during admission could transiently affects the measurements.

The study protocol was approved by the Institutional Review Board of the Kobe University Graduate School of Medicine (No. B250070), and the requirement for informed consent was waived due to the retrospective design of the study. An opt-out option was provided for all patients. The study was conducted in accordance with the principles of the Declaration of Helsinki.

### 2.2 Statistical analysis

Two analytical approaches were used to compare the eGFR slopes before and after initiating SGLT2 inhibitor therapy. First, individual eGFR slopes of each patient were calculated using ordinary least-squares linear regression, and pre- and posttreatment slopes were compared using the Wilcoxon signed-rank test. Second, a linear mixed-effects model was employed to evaluate the difference in eGFR slopes. In this model, eGFR was the dependent variable, while time since initiation of SGLT2 inhibitor therapy, treatment phase (pre- vs. post-initiation), and their interaction were the fixed effects (unadjusted model). The regression coefficient for time represented the eGFR slope before treatment, and the sum of the coefficients for time and the interaction term represented the eGFR slope after treatment.

The initial eGFR decline following SGLT2 inhibitor therapy was evaluated by assessing the statistical significance of the intercept in the linear mixed-effects model of eGFR slope. Additionally, the immediate change in eGFR before and after initiating SGLT2 inhibitor therapy was calculated, and differences between patients with and without FD were compared using the Mann–Whitney *U* test.

For UACR and plasma BNP levels, geometric mean changes over the 1-year periods before and after initiating SGLT2 inhibitor therapy were compared using a linear mixed-effects model. In this model, log-transformed UACR or BNP was the dependent variable, with SGLT2 inhibitor treatment status (pre- vs. post-initiation) being the fixed effect (unadjusted model). The exponentiated regression coefficient for SGLT2 inhibitor use was interpreted as the geometric mean ratio, representing the relative change in UACR or BNP levels following treatment.

To account for potential confounding factors, linear mixed-effects models were created with three models of adjustment. Model 1 included age, sex, and baseline values of eGFR, UACR, or BNP levels. Model 2 was Model 1 + ERT and pharmacological chaperone therapy. Model 3 included all covariates from Model 2 along with the prescribed doses of ACE-Is, ARBs, and angiotensin receptor–neprilysin inhibitor. Furthermore, the association between eGFR slopes before and after SGLT2 inhibitor therapy was adjusted for the severity of FD (Model 4). The severity was assessed using a weighted score [10].

Baseline characteristics between the FD and non-FD groups were compared using the unpaired *t*-test for continuous variables and Fisher's exact test for categorical variables.

A two-sided *P*-value of < 0.05 was considered statistically significant. All statistical analyses were performed using the Stata/MP 14.2 software for Windows (Stata, College Station, TX, USA).

## 3 Results

### 3.1 Patients' characteristics

The patients' baseline characteristics are shown in Table 1. Age and sex were comparable between the FD and non-FD group, and none of the patients in either group had diabetes mellitus. In the FD group, no patients had hypertension or dyslipidemia, whereas in the non-FD group, 14 patients had hypertension and 8 had dyslipidemia. Mean eGFR was significantly higher in the FD group than in the non-FD group. The use of RAS inhibitors did not differ between groups.

**Table 1:** Patient's characteristics at baseline.

	Fabry (*N* = 10)	Non-Fabry (*N* = 18)	P
Age (years)	41 ± 18	46 ± 13	0.397
Male (n, %)	7 (70)	10 (56)	0.689
Hypertension (n, %)	0 (0)	14 (78)	<0.001
Diabetes mellitus (n, %)	0 (0)	0 (0)	–
Hyperlipidemia (n, %)	0 (0)	8 (44)	0.004
Genetic type (n, %)			
Classical	10 (100)	–	–
Late onset	0 (0)	–	–
Weighted score (%)	205 [160–285]	NA	–
eGFR (mL/min/1.73 m^2^)	87.9 ± 33.5	61.2 ± 12.2	0.005
UACR (mg/gCr)	91.1 [65.0–420.9]	NA	–
BNP (pg/mL)	39.1 [11.3–288.3]	NA	–
αGAL-A activity (nmol/mg protein/h)	0.4 [0.1–5.8]	NA	–
ERT / Chaperone therapy (n, %)			
Agalsidase-alpha	5 (50)	–	–
Agalsidase-beta	3 (30)	–	–
Migalastat	1 (10)	–	–
None	1 (10)	–	–
RAS inhibitors (n, %)			
Enalapril	2 (20)	0 (0)	0.119
Perindopril	1 (10)	0 (0)	0.357
Losartan	2 (20)	9 (50)	0.226
Olmesartan	1 (10)	3 (17)	1.000
Candesartan	0 (0)	3 (17)	0.533
Azilsartan	0 (0)	2 (11)	0.524
Irbesartan	0 (0)	1 (6)	1.000
Sacubitril/Valsartan	1 (10)	0 (0)	0.357

eGFR, estimated glomerular filtration rate; UACR, urinary albumin-to-creatinine ratio; BNP, brain natriuretic peptide; αGAL-A, alfa-galactosidase A; ERT, enzyme replacement therapy; RAS, renin-angiotensin system.

In the FD group, all patients exhibited the classical phenotype. The median UACR was 91.1 g/gCr (interquartile range [IQR], 65.0–420.9). With respect to Fabry-specific therapies, five patients received agalsidase-alpha, three received agalsidase-beta, and one received migalastat. Regarding SGLT2 inhibitors, dapagliflozin was administered to nine patients and empagliflozin to one patient. None of the Fabry patients received mineralocorticoid receptor antagonists or vitamin D analogs.

In the non-FD group, the underlying kidney diseases included IgA nephropathy in 12 patients (66.7 %), nephrosclerosis in 1 (5.6 %), focal segmental glomerulosclerosis in 1 (5.6 %), membranous nephropathy in 1 (5.6 %), Alport syndrome in 1 (5.6 %), and unknown causes in 2 patients (11.1 %).

### 3.2 Changes in eGFR following SGLT2 inhibitor therapy

**Fig. 2:**
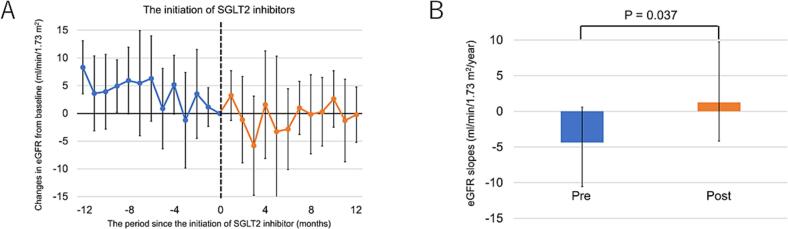
(A) Change in eGFR from baseline before and after initiating SGLT2 inhibitor therapy in patients with FD. (B) Comparison of eGFR slope before and after initiating SGLT2 inhibitor therapy in patients with FD (Least-squares method).

**Fig. 3:**
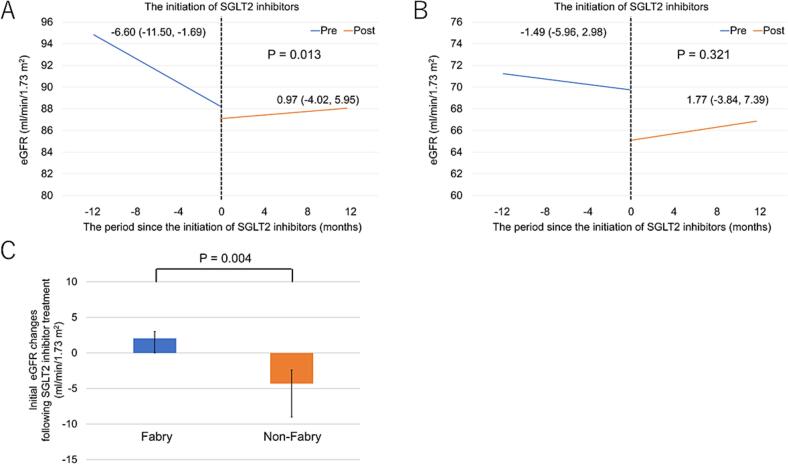
(A) Comparison of eGFR slope before and after initiating SGLT2 inhibitor therapy in patients with FD (a linear mixed-effects model). (B) Comparison of eGFR slope before and after initiating SGLT2 inhibitor therapy in patients with CKD without FD (a linear mixed-effects model). (C) Comparison of the immediate change in eGFR before and after initiating SGLT2 inhibitor therapy in patients with and without FD.

Fig. 2A presents the trajectory of the eGFR before and after initiating SGLT2 inhibitor therapy. A declining trend in eGFR was noted before treatment, but the rate of decline seemed to slow after initiating treatment. In the ordinary least-squares linear regression analysis, the annual rate of eGFR decline significantly improved from −4.38 (IQR, −10.57 to 0.59) to 1.25 (IQR, −4.16 to 9.74) mL/min/1.73 m^2^/year (Fig. 2B). Additionally, the linear mixed-effects model also showed a comparable attenuation in eGFR decline (Fig. 3A) that remained statistically significant after adjusting for potential confounders (Table 2).

**Table 2:** Estimated GFR slopes and 95 % confidence intervals before and after SGLT2 inhibitor therapy in multivariate linear mixed-effects models.

	eGFR slope (mL/min/1.73 m^2^/year)		P
	Before SGLT2 inhibitors	After SGLT2 inhibitors	
Unadjusted	−6.60 (−11.50, −1.69)	0.97 (−4.02, 5.95)	0.013
Model 1	−6.32 (−11.10, −1.53)	0.78 (−4.07, 5.62)	0.020
Model 2	−5.59 (−10.53, −0.66)	1.03 (−4.02, 6.08)	0.031
Model 3^a^	−6.92 (−12.44, −1.40)	1.95 (−3.52, 7.43)	0.011
Model 4^a^	−7.01 (−12.48, −1.53)	1.61 (−3.88, 7.10)	0.014

eGFR, estimated glomerular filtration rate; SGLT2, sodium–glucose co-transporter 2.

a The number of patients with Fabry disease was nine.

The rate of eGFR decline before initiating SGLT2 inhibitor therapy was more rapid in patients with FD than in those without (Fig. 3A, B). Although the change in the rate of eGFR decline before and after initiating SGLT2 therapy did not reach statistical significance, it tended to improve in patients without FD. After adjusting for age, sex, baseline eGFR, and use of RAS inhibitors, a similar trend was noted, with the eGFR slope changing from −0.79 (95 % confidence interval [CI]: −3.39 to 1.81) to 0.11 (95 % CI: −2.94 to 3.17) mL/min/1.73 m^2^/year.

Moreover, a significant initial decline in eGFR was noted after initiating SGLT2 inhibitor therapy in patients without FD, which was not observed in patients with FD (Table 3). Notably, the magnitude of the initial eGFR decline was significantly greater in patients without FD than in those with FD (Fig. 3C). In the analysis of the non-FD group matched to the FD group for age, sex, and baseline eGFR (*N* = 6; non-FD vs. FD: age, 39 ± 17 vs. 41 ± 18 years, *P* = 0.889; male, 67 % vs. 70 %, *P* = 1.00; eGFR, 73.8 ± 10.8 vs. 87.9 ± 33.5 mL/min/1.73 m^2^, *P* = 0.218), a significant initial decline in eGFR after SGLT2 inhibitor therapy was still observed (Supplementary Fig. 1).

**Table 3:** Initial eGFR changes and 95 % confidence intervals following SGLT2 inhibitor therapy in patients with and without Fabry disease: multivariate linear mixed-effects model analysis.

	Fabry (N = 10)		Non-Fabry (N = 18)	
	Initial eGFR changes (mL/min/1.73 m^2^)	P	Initial eGFR changes (mL/min/1.73 m^2^)	P
Unadjusted	−1.15 (−4.33, 2.04)	0.480	−3.50 (−5.50, −1.50)	0.001
Model 1	−1.28 (−4.46, 1.91)	0.432	−3.59 (−5.57, −1.60)	<0.001
Model 2	−1.53 (−4.72, 1.66)	0.347	−3.59 (−5.57, −1.60)	<0.001
Model 3^a^	−1.54 (−4.96, 1.88)	0.377	−3.56 (−5.52, −1.60)	<0.001

eGFR, estimated glomerular filtration rate.

a The number of patients with Fabry disease was nine.

### 3.3 Changes in urinary albumin excretion and plasma BNP levels following SGLT2 inhibitor therapy

**Fig. 4:**
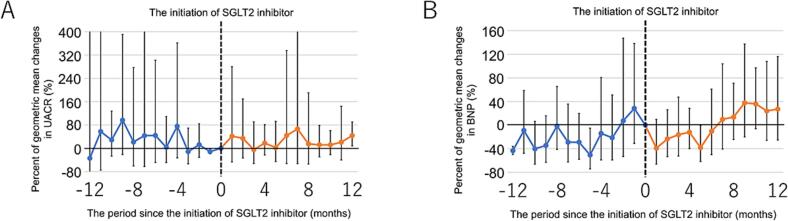
(A) Geometric mean changes in UACR from baseline before and after initiating SGLT2 inhibitor therapy in patients with FD. (B) Geometric mean changes in BNP levels from baseline before and after initiating SGLT2 inhibitor therapy in patients with FD.

Fig. 4A shows the geometric mean changes in UACR from baseline before and after initiating SGLT2 inhibitor therapy, demonstrating that UACR remained stable. In the linear mixed-effects model analysis, UACR values after initiating therapy were comparable to pretreatment values in both unadjusted and adjusted models (Table 4).

**Table 4:** Geometric mean changes and 95 % confidence intervals in UACR and plasma BNP levels following SGLT2 inhibitor therapy in multivariate linear mixed-effects models.

	UACR (%)	P	BNP (%)	P
Unadjusted	20.2 (−4.2, 50.7)	0.111	0.5 (−14.0, 17.5)	0.948
Model 1	21.4 (−3.4, 52.6)	0.096	−11.8 (−27.7, 7.7)	0.218
Model 2	26.2 (−2.6, 53.5)	0.083	−2.2 (−17.0, 15.3)	0.792
Model 3^a^	13.8 (−8.9, 26.9)	0.360	−20.3 (−38.5, 3.2)	0.085

UACR, urinary albumin-to-creatinine ratio; BNP, brain natriuretic peptide; SGLT2, sodium–glucose co-transporter 2.

a The number of patients with Fabry disease was nine.

Similarly, plasma BNP levels remained unchanged following SGLT2 inhibitor therapy (Fig. 4B). After adjusting for potential confounders, the linear mixed-effects model analysis confirmed that posttreatment plasma BNP levels did not notably change (Table 4).

## 4 Discussion

Our study revealed that treatment with SGLT2 inhibitors attenuated the decline in eGFR in patients with FD, similar to patients with non-FD CKD [11,12]. However, unlike patients without FD, those with FD did not experience a significant initial decline in eGFR following treatment initiation. Moreover, SGLT2 inhibitor therapy was not related to significant reductions in albuminuria and plasma BNP levels in the FD cohort.

The renoprotective effects of SGLT2 inhibitors, particularly slowing the decline in eGFR, have been established in large trials involving patients with CKD with and without diabetes mellitus [[11], [12], [13], [14]]. However, subgroup analyses of these trials revealed that the magnitude of this benefit may differ according to the underlying etiology of CKD [[15], [16], [17]]. Among patients with nondiabetic CKD, SGLT2 inhibitors have favorable effects in IgA nephropathy, whereas their efficacy is limited in focal segmental glomerulosclerosis and uncertain in autosomal dominant polycystic kidney disease and autoimmune-related nephritis. To the best of our knowledge, the efficacy of SGLT2 inhibitors in patients with FD has not been demonstrated in observational studies or randomized controlled trials. An observational cohort study in the UK reported that none of the FD patients who experienced cardiovascular or renal outcomes had received SGLT2 inhibitors [18]. Furthermore, aside from ERT and chaperone therapy, there are no established supportive therapies for kidney involvement in FD. Therefore, our study is the first to suggest that SGLT2 inhibitors may attenuate eGFR decline in patients with FD. Similar to other kidney diseases, preserving kidney function is essential in FD. As cardiac involvement is common in FD, preserving kidney function may also carry significant implications from a cardiorenal perspective.

On the other hand, in our study, treatment with SGLT2 inhibitors did not reduce albuminuria in patients with FD, in contrast to findings from previous large-scale clinical trials in patients with CKD and small case series in those with FD [[19], [20], [21]]. This discrepancy may be partly attributable to results indicating that the albuminuria-reducing effect of SGLT2 inhibitors is more remarkably attenuated in patients without diabetes compared to those with diabetes [19,20]. Additionally, reduction in albuminuria does not occur in all patients treated with SGLT2 inhibitors, with a reporting that 42.3 % of patients with diabetes failed to show a decrease in UACR despite treatment [22]. Thus, a larger sample size may be needed to detect a statistically significant effect on albuminuria in our cohort. Indeed, previous small-scale studies involving patients with nondiabetic CKD have also failed to report reductions in proteinuria following short-term treatment [23,24]. Furthermore, a retrospective international multicenter study that evaluated the effects of SGLT2 inhibitors in patients with biopsy-proven glomerulonephritis reported that 31 % of patients had insufficient reduction in proteinuria [25]. These results collectively underscore the need for larger and longer-term studies to confirm whether SGLT2 inhibitors can effectively reduce albuminuria in patients with FD.

An initial decline in eGFR commonly occurs after initiating SGLT2 inhibitor therapy [19,20]. However, in our study, no significant initial decline in eGFR was noted in patients with FD. The magnitude of this acute eGFR decline seems to vary among individuals. In a previous study, 40.9 % of patients showed no initial eGFR decline, 37.6 % experienced a decline of 0–10 %, and only 21.5 % showed a decline >10 % [22]. Moreover, as the initial decline in eGFR correlates strongly with a reduction in albuminuria [19,22], patients who fail to show a decrease in albuminuria, such as those with FD in our study, may also be less likely to experience an early decline in eGFR. However, there is no clear correlation between the magnitude of the initial eGFR decline and long-term improvements in the eGFR slope [26]. The initial dip also tends to be less pronounced in patients without diabetes or in those with an already compromised hemodynamic status, such as in elderly individuals or those with concomitant heart failure; patients with FD are presumed to share similar characteristics. Post hoc analyses of the DAPA-CKD trial revealed that a slower decline in eGFR was noted regardless of the presence or absence of an initial eGFR decline [26], suggesting that the renoprotective effects of SGLT2 inhibitors can be expected even in cases wherein an initial decline in eGFR does not occur.

There are several possibilities responsible for the underlying mechanisms regarding the renoprotective effects of SGLT2 inhibitors in FD. In our study, the initial decline in eGFR after initiating SGLT2 inhibitor therapy was smaller in patients with FD compared to those with other forms of CKD, indicating that hemodynamic changes, such as reduced intraglomerular pressure, may play a limited role in FD. Hence, it is hypothesized that nonhemodynamic mechanisms are primarily responsible for the renoprotective effects of SGLT2 inhibitors in FD. Inflammation and oxidative stress play a role in the pathogenesis of organ damage in FD [27,28]. SGLT2 inhibitors reportedly reduce oxidative stress by inhibiting glucose reabsorption and suppressing proinflammatory cytokines such as interleukin-6 and tumor necrosis factor-alpha [29]. Additionally, SGLT2 inhibitors suppress renal fibrosis markers, including transforming growth factor-beta 1 [30]. Therefore, it is plausible that SGLT2 inhibitors may attenuate the progression of renal fibrosis and structural glomerular damage in FD by mitigating oxidative stress and inflammation, slowing the decline in eGFR. Another possible mechanism involves the improvement of oxygen supply-demand balance in renal tubular cells. Accumulation of Gb3 increases oxidative stress and induces mitochondrial dysfunction, which may be a source of reactive oxygen species [31]. By inhibiting sodium reabsorption, SGLT2 inhibitors reduce ATP consumption in proximal tubular cells, reducing oxygen demand, which may improve tubulointerstitial hypoxia and reduce mitochondria stress [32]. Consequently, this mechanism may also suppress the progression of tubular atrophy and interstitial fibrosis, thereby contributing to the renoprotective effects of SGLT2 inhibitors in patients with FD.

In this study, plasma BNP levels remained unchanged after SGLT2 inhibitor therapy. In contrast, previous clinical trials in patients with heart failure demonstrated that SGLT2 inhibitors reduce cardiovascular death, hospitalization for heart failure, and plasma NT-proBNP levels [33,34]. A plausible explanation for the discrepancy is that most of our patients had no history of heart failure and preserved cardiac function. As improved cardiac function has been reported to enhance renal function [35], the cardioprotective effects of SGLT2 inhibitors may contribute to renoprotection. Nevertheless, our study demonstrated the renoprotective effects of SGLT2 inhibitors in patients with FD, despite unchanged plasma BNP levels, suggesting that these benefits may occur independently of improvements in cardiac function.

This study has several limitations. First, the sample size was relatively small. However, given that FD is a rare disease, the limited number of patients in a single-center study was considerable. To mitigate the potential bias associated with the small sample size, we employed linear mixed-effects models in our statistical analysis. Ideally, future studies with larger, multicenter cohorts are needed to validate our findings. Second, we were unable to evaluate the effects of SGLT2 inhibitors according to the genotypic heterogeneity of FD. Over 1000 genetic variants have been reported in FD, making genotype-specific analyses extremely challenging. Nevertheless, all patients with FD in our study exhibited the classical phenotype, which is typically associated with more severe organ involvement. Therefore, demonstrating the potential efficacy of SGLT2 inhibitors in this high-risk population is of clinical significance. Further studies are warranted to explore their therapeutic effects in patients with the late-onset phenotype. Finally, the observational period of this study was relatively short, which may limit the evaluation of the long-term kidney outcomes. As the use of SGLT2 inhibitors in CKD has only recently become more widespread, acquiring long-term follow-up data remains challenging. Although traditional assessments of kidney disease progression have relied on hard endpoints observed over extended periods, recent nephrology research has increasingly adopted the eGFR slope as a surrogate marker. We employed this approach to evaluate treatment effects within the constraints of the available follow-up period.

Currently, a multicenter prospective observational cohort study is ongoing to evaluate the effects of dapagliflozin in patients with FD and CKD stages 1 to 3 [36]. This may help address some of the limitations of our analysis.

## 5 Conclusions

In conclusion, SGLT2 inhibitors may be a novel therapeutic option to slow kidney function decline in this population. Further evaluation in prospective studies with larger sample sizes are warranted to confirm our findings.

## Funding

This research did not receive any specific grant from funding agencies in the public, commercial, or not-for-profit sectors.

## CRediT authorship contribution statement

**Hayaki Okamoto:** Writing – original draft. **Shunsuke Goto:** Formal analysis. **Mika Fujita:** Data curation. **Hideki Fujii:** Writing – review & editing.

## Declaration of competing interest

H.F. has received lecture fees from Sumitomo Pharma, Takeda Pharmaceutical Company, Amicus Therapeutics, and Sanofi, and research support from JCR Pharma.

## Data Availability

Data will be made available on request.

## Appendix A Supplementary data

Supplementary material Image 1
