# Supplementary material for: Renoprotective effects of SGLT2 inhibitors in patients with Fabry disease
Source: Mol Genet Metab Rep. 2025 Oct 17;45:101271. doi: 10.1016/j.ymgmr.2025.101271 (PMC12554905; doi:10.1016/j.ymgmr.2025.101271)
Supplement: Supplementary file 1 — Supplementary material [file mmc1.docx]

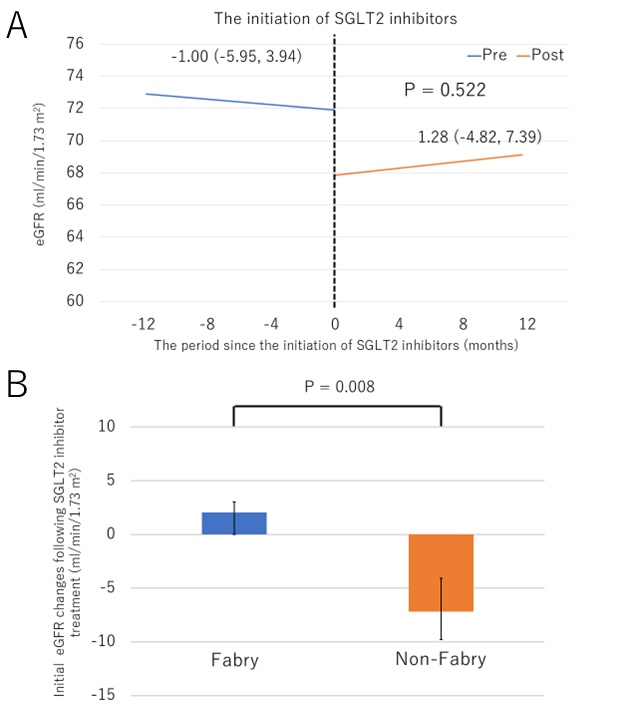


**Supplementary Figure 1.** (A) Comparison of eGFR slope before and after initiating SGLT2 inhibitor therapy in the non-Fabry disease (FD) group (N = 6) matched to the FD group for age, sex, and baseline eGFR (a linear mixed-effects model). (B) Comparison of the immediate change in eGFR before and after initiating SGLT2 inhibitor therapy in the non-FD group matched to the FD group for age, sex, and baseline eGFR.
